# Hexokinase 2 promotes tumor growth and metastasis by regulating lactate production in pancreatic cancer

**DOI:** 10.18632/oncotarget.9760

**Published:** 2016-06-01

**Authors:** Marybeth Anderson, Raoud Marayati, Richard Moffitt, Jen Jen Yeh

**Affiliations:** ^1^ Curriculum in Genetics & Molecular Biology, The University of North Carolina, Chapel Hill, NC; ^2^ Lineberger Comprehensive Cancer Center, The University of North Carolina, Chapel Hill, NC; ^3^ Departments of Surgery and Pharmacology, The University of North Carolina, Chapel Hill, NC

**Keywords:** pancreatic cancer, hexokinase 2, glycolysis, metastasis

## Abstract

Pancreatic ductal adenocarcinoma (PDAC) is a *KRAS*-driven cancer with a high incidence of metastasis and an overall poor prognosis. Previous work in a genetically engineered mouse model of PDAC showed glucose metabolism to be important for maintaining tumor growth. Multiple glycolytic enzymes, including hexokinase 2 (HK2), were upregulated in primary PDAC patient tumors, supporting a role for glycolysis in promoting human disease. HK2 was most highly expressed in PDAC metastases, suggesting a link between HK2 and aggressive tumor biology. In support of this we found HK2 expression to be associated with shorter overall survival in PDAC patients undergoing curative surgery. Transient and stable knockdown of HK2 in primary PDAC cell lines decreased lactate production, anchorage independent growth (AIG) and invasion through a reconstituted matrix. Conversely, stable overexpression of HK2 increased lactate production, cell proliferation, AIG and invasion. Pharmacologic inhibition of lactate production reduced the HK2-driven increase in invasion while addition of extracellular lactate enhanced invasion, together providing a link between glycolytic activity and metastatic potential. Stable knockdown of HK2 decreased primary tumor growth in cell line xenografts and decreased incidence of lung metastasis after tail vein injection. Gene expression analysis of tumors with decreased HK2 expression showed alterations in VEGF-A signaling, a pathway important for angiogenesis and metastasis, consistent with a requirement of HK2 in promoting metastasis. Overall our data provides strong evidence for the role of HK2 in promoting PDAC disease progression, suggesting that direct inhibition of HK2 may be a promising approach in the clinic.

## INTRODUCTION

Pancreatic ductal adenocarcinoma (PDAC) is a highly lethal malignancy with a five-year overall survival of <6% [1]. Over half of all patients initially present with metastatic disease, where treatment options are limited to cytotoxic chemotherapies that are not well tolerated and provide modest improvements in overall survival [2]. Over 90% of PDAC tumors contain activating mutations in the oncogene *KRAS*, suggesting that it may be an ideal target for therapy [3]. While knockdown of KRAS inhibits PDAC cell growth *in vitro*, direct targeting of KRAS and its main effector pathways have proven unsuccessful in the clinic [3, 4]. Much work has focused on identifying additional pathways promoting *KRAS* driven tumor growth in PDAC, with the hope of identifying new targets for therapy.

The importance of glucose metabolism in *KRAS* driven oncogenesis is well recognized [4–7]. In a genetically engineered mouse model (GEMM) of PDAC, oncogenic Kras activity promoted transcriptional upregulation of key enzymes involved in glucose processing, including those regulating glycolysis, hexosamine biosynthesis and the pentose phosphate pathway [8]. Activity of these enzymes was required for tumor growth, suggesting a role for targeting glucose uptake and anabolism in PDAC [8]. Hexokinase 2 (HK2) is an enzyme responsible for phosphorylating glucose, a reaction necessary for glucose processing [9, 10]. Four hexokinase isoforms (HK1-HK4) are expressed at varying levels in tissues, but HK2 is the sole isoform overexpressed in cancer [11–15]. Genetic deletion of *Hk2* caused a decrease in tumor burden and increased overall survival of *Kras*-driven lung and *ErbB2*-driven breast cancer GEMMS [12]. In addition, HK2 knockdown has been found to successfully inhibit tumor growth in glioblastoma, medulloblastoma and renal cell carcinoma [16–18].

While a direct role for HK2 in PDAC has yet to be reported, studies examining gene expression and PDAC patient outcomes have shown an association between increased expression of HK2 and more aggressive disease [19] [20]. Anabolic glucose metabolism promoted disease progression in a PDAC GEMM, however analysis of human tissue revealed increased expression of genes involved in aerobic glycolysis, including HK2, in primary PDAC and PDAC metastases without changes in expression of anabolic genes, suggesting that glycolysis may be important in human disease [20]. PDAC cell lines with elevated rates of glycolysis showed increase expression of an epithelial-mesenchymal transition (EMT) gene signature and were classified as a quasi-mesenchymal, a PDAC subtype previously associated with shorter overall survival [21–23]. Taken together these studies provide strong, indirect evidence suggesting a role for HK2 and glycolysis in promoting PDAC disease progression.

The current study shows that HK2 is required for primary tumor growth and metastasis in PDAC. By overexpressing HK2 in PDAC cell lines, we show that increased levels of HK2 are sufficient to promote cell proliferation, anchorage independent growth (AIG) and invasion, supporting a role for HK2 in driving disease progression. Pharmacologic inhibition of lactate production dampens the effects of HK2 on invasion while increased extracellular lactate is sufficient to promote invasion. Overall, this study provides a mechanistic link between HK2 and metastasis via regulation of lactate production and suggests that direct inhibition of HK2 may be a promising approach for treating PDAC.

## RESULTS

### Genes involved in glucose uptake and glycolysis are dysregulated in PDAC

We examined the expression of genes involved with glucose metabolism using a previously described dataset of tumors from primary and metastatic sites of 143 PDAC patients (GSE 71729) [24]. A list of 153 unique genes of interest was compiled using existing KEGG and Reactome gene lists for glucose metabolism, glycolysis and gluconeogenesis, the pentose phosphate pathway and O-glycan biosynthesis (Supplementary Table S1) [25]. Also included were 14 genes belonging to the family of sugar transport facilitators (SLC2A/GLUT) that are responsible for glucose uptake [26]. To identify genes associated with tumorigenesis, we looked for those highly expressed in primary tumors compared to unmatched normal pancreas. To identify genes associated with metastasis, we looked for genes highly expressed in metastases compared to primary tumors.

**Figure 1:**
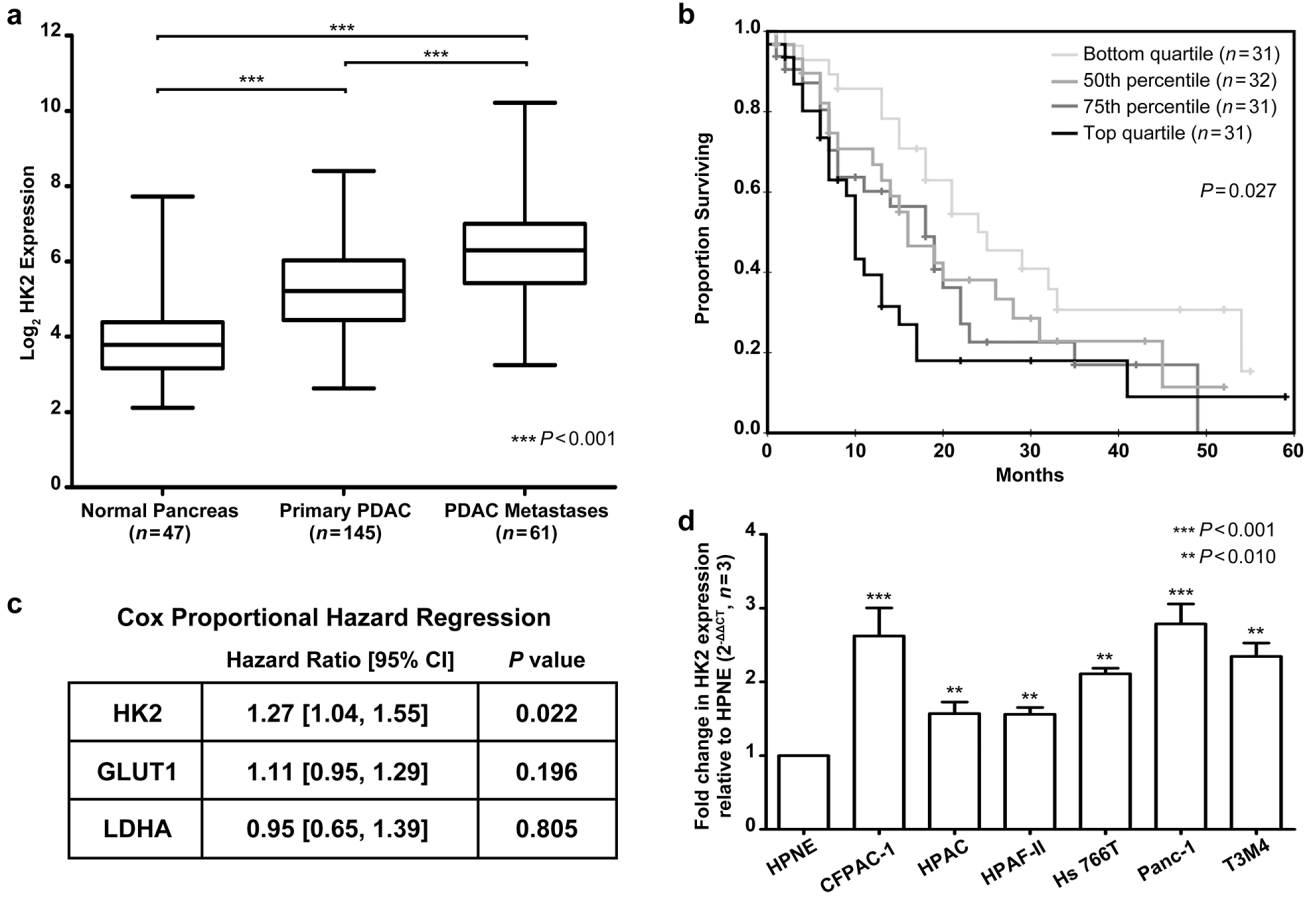
HK2 expression is upregulated in PDAC and associated with a poor overall survival **a.** Log_2_ expression of HK2 in normal pancreas, primary PDAC, and PDAC metastases. Box shows median expression with upper and lower quartiles and whiskers show maximum and minimum values. A one-way ANOVA with Bonferroni correction for multiple comparisons test determined statistical significance. **b.** Groups for Kaplan Meier survival analysis were based off HK2 expression in primary tumors. Lowest quartile showed median survival of 24 months (95% CI [14, 34]) while highest quartile showed median survival of 10 months (95% CI [9, 11]). **c.** Correlation between HK2, GLUT1 and LDHA expression in primary tumors (*n*=125) and overall survival as determined by univariate Cox proportional regression. Hazard ratios and 95% CI shown. **d.** Fold change in HK2 expression across a panel of PDAC cell lines relative to the immortalized epithelial cell line HPNE. Fold change determined using the ΔΔCT method with mean and standard error of the mean (SEM) shown (*n*=3 technical replicates).

Four genes – glucose transporter 1 (GLUT1), HK2, lactate dehydrogenase A (LDHA), and triosephosphate isomerase 1 (TPI1) – were upregulated in primary tumors compared to normal pancreas and in metastases compared to primary tumors (*P*<0.001, Figure 1a, Supplementary Figure S1). GLUT1 and HK2 both play a role in glucose uptake. LDHA is a key enzyme responsible for producing lactate from pyruvate, the final step in aerobic glycolysis [27, 28]. GLUT1, HK2, and LDHA were previously found to be regulated in an oncogenic Kras dependent manner, suggesting that they may be important for KRAS-driven tumor growth [8]. TPI1 catalyzes an isomerization reaction in the glycolytic cascade but is not regulated in a Kras-dependent manner and was, therefore, not further studied [8].

### Increased HK2 expression is associated with poor patient survival after surgery

Increasing expression of key enzymes regulating glucose uptake and glycolysis in primary tumors and metastases suggest that these pathways are associated with aggressive tumor biology. To determine if GLUT1, LDHA, or HK2 was associated with clinical outcome, we evaluated the relationship between gene expression and patient survival (Figure 1). Patients with tumors containing high HK2 expression had a median survival of 13 months while patients with tumors containing low HK2 expression had a median survival of 21 months (*P*=0.027, Figure 1b). HK2 was also associated with shorter overall survival in patients with localized tumors who underwent curative surgery (hazard ratio (HR) 1.31 (1.07, 1.60), Figure 1c), suggesting that high HK2 expression may be associated with early disease relapse and metastasis. Neither GLUT1 nor LDHA expression was associated with patient survival (Figure 1c).

### HK2 is necessary for AIG and invasion in PDAC cell lines

**Figure 2:**
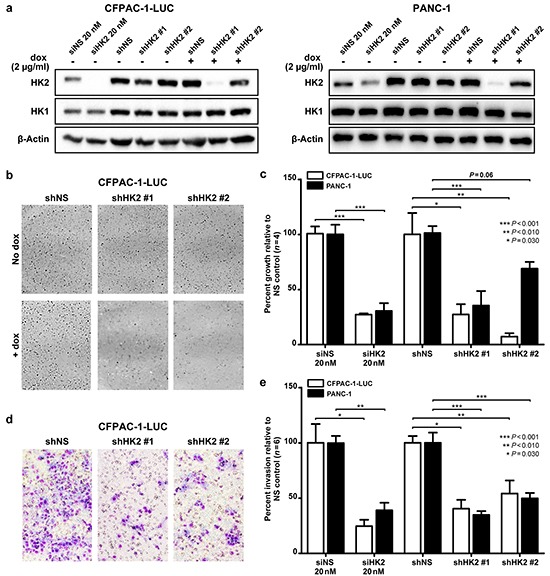
HK2 is required for AIG and invasion in PDAC cell lines **a.** Transient and stable knockdown of HK2 was achieved using siRNA (siHK2, 20 nM) and a doxycycline inducible lentiviral shRNA construct (shHK2# and #2) in CFPAC-1-LUC and PANC-1. Cells were isolated after 72 hours of doxycycline (2 μg/mL) exposure. **b.** Representative images of colony formation in soft agar assays. **c.** Percent of growth with HK2 knockdown relative to control (siNS or shNS). Mean ± SEM of biological replicates (*n*=4) shown with student's t-tests for statistical significance. **d.** Representative images from Matrigel coated transwell invasion assays. **e.** Percent invasion of HK2 knockdown relative to control (siNS or shNS). Mean ± SEM of biological replicates (*n*=6) shown with student's t-tests for statistical significance.

To directly assess the requirement of HK2 for promoting tumor growth and invasion, si- and shRNA were used to transiently and stably knockdown HK2 in two PDAC cell lines, CFPAC-1-LUC and PANC-1 (Figure 2a). Both lines contain activating mutations in *KRAS* and exhibit the highest levels of HK2 expression relative to the normal immortalized epithelial cell line HPNE across a panel of PDAC cell lines (*P*<0.001, Figure 1d). Levels of HK1 were unaffected by transient and stable knockdown of HK2, suggesting that our constructs were HK2 specific and that there was no compensatory increase in HK1 expression (Figure 2a).

To examine the effect of HK2 knockdown on PDAC cell growth, a soft agar assay was used to assess for AIG, a phenotype associated with both tumor growth and metastatic potential [29] (Figure 2b). Transient knockdown of HK2 in the CFPAC-1-LUC cell line caused a 72.7% decrease in colony growth compared to that observed for the nonspecific (NS) control (*P*<0.001, Figure 2c). Similar findings were observed with stable knockdown of HK2 (*P*<0.030, Figure 2c). Transient and stable knockdown in PANC-1 with siHK2 and shHK2#1 resulted in an 82.0% and 71.0% decrease in colony growth relative to NS, respectively (*P*<0.001, Figure 2c). Stable knockdown using shHK2#2 caused 30.1% growth inhibition relative to shNS (*P*=0.06, Figure 2c). The dampened effect on colony growth may be explained by inefficient knockdown of HK2 with the shHK2#2 construct (Figure 2a). We hypothesize that the decrease in AIG observed results from a decrease in cell proliferation with HK2 knockdown, as decreased anchorage dependent growth was observed in both cell lines with transient and stable knockdown of HK2 using a MTT assay (Supplementary Figure S2). To assess the effect of HK2 knockdown on invasion, a Matrigel coated transwell invasion assay was used (Figure 2d). Transient and stable knockdown of HK2 caused an approximate 50% decrease in invasion in both cell lines (*P*<0.030, Figure 2e). These results show that HK2 is required for PDAC AIG and invasion.

### HK2 is sufficient to promote AIG and invasion in PDAC cell lines

**Figure 3:**
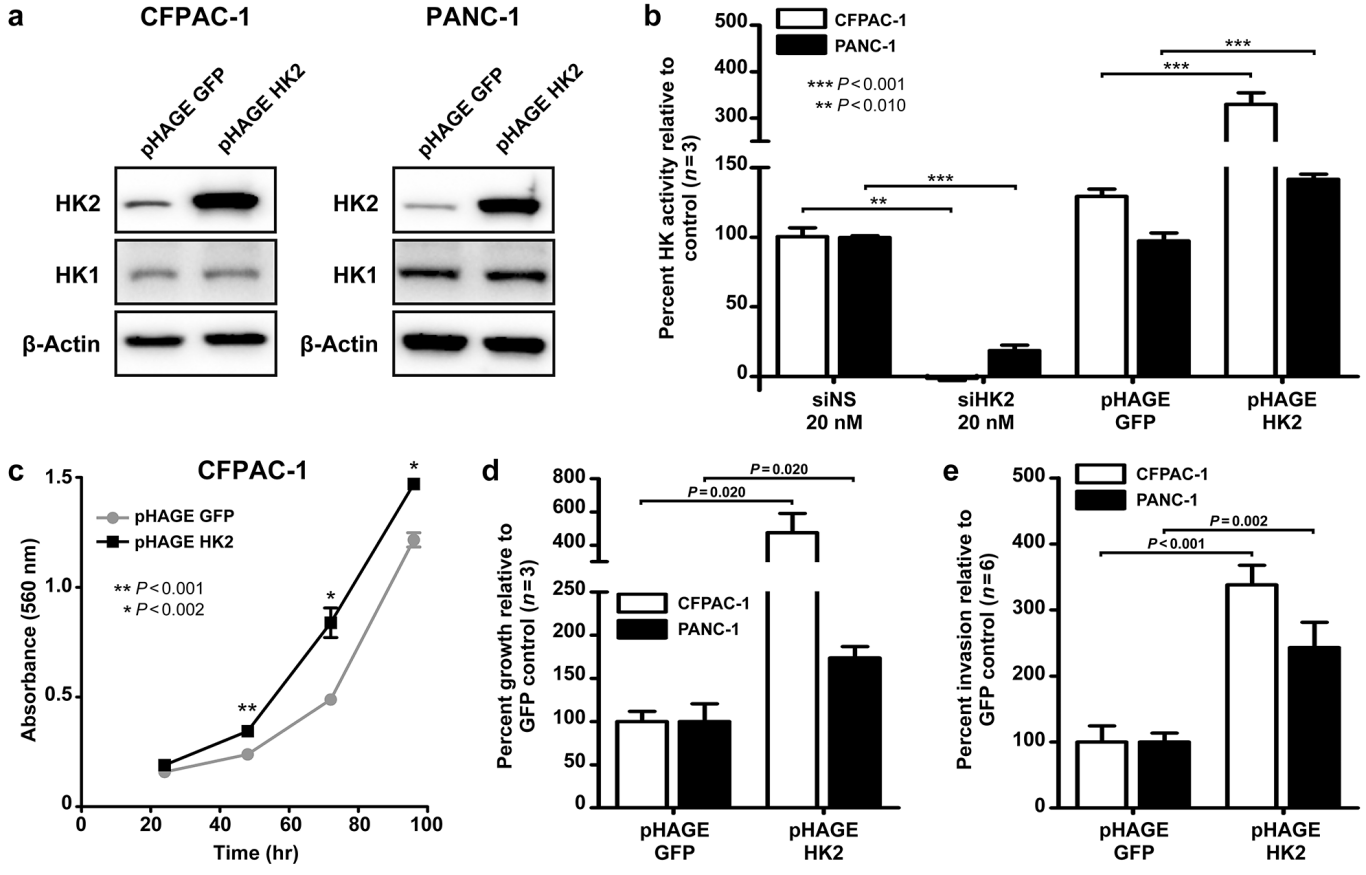
HK2 is sufficient to promote AIG and invasion in PDAC cell lines **a.** Stable overexpression of HK2 (pHAGE HK2) relative to control (pHAGE GFP) in CFPAC-1 and PANC-1 cell lines. **b.** Percent hexokinase activity of knockdown (siNS vs. siHK2) and overexpression (pHAGE GFP vs. pHAGE HK2) cell lines relative to control. Mean ± SEM of biological replicates (*n*=3) shown with student's t-tests for statistical significance. **c.** Cell proliferation in CFPAC-1-GFP and CFPAC-1-HK2 as determined using a MTT assay. Mean ± SEM of technical replicates (*n*=4) shown with student's t-tests for statistical significance at 48, 72, and 96 hours of growth. **d.** Percent colony growth with HK2 overexpression relative to control. Mean ± SEM of biological replicates (*n*=3) shown with student's t-tests for statistical significance. **e.** Percent invasion with HK2 overexpression relative to control. Mean ± SEM of biological replicates (*n*=6) shown with student's t-tests for statistical significance.

HK2 expression is limited in most normal tissues but preferentially upregulated in cancer cells [11]. Whether HK2 is sufficient to promote PDAC growth and metastasis is unknown. To examine this, two PDAC cell lines stably overexpressing HK2 cDNA were generated (CFPAC-1-HK2 and PANC-1-HK2, Figure 3). Hexokinase (HK) activity was measured to confirm that stable HK2 overexpression resulted in increased protein function. A 3.3-fold increase in HK activity was observed in CFPAC-1-HK2 and a 1.4-fold increase was observed for PANC-1-HK2 (*P*<0.001, Figure 3b). Additionally transient knockdown of HK2 with siRNA caused a significant decrease in HK when compared to the control (*P*<0.010, Figure 3b). No change in HK1 expression was found in the cell lines generated, suggesting that the increased HK activity can be solely attributed to changes in the level of HK2.

We next determined the effects of HK2 overexpression on AIG and invasion. A 5.1-fold increase in colony growth was observed with stable HK2 overexpression relative to the GFP control in CFPAC-1-HK2, while a 1.7-fold increase was observed in PANC-1-HK2 (*P*=0.020, Figure 3d). We hypothesized that this increase in AIG results from an increased rate of cell proliferation, as HK2 overexpression caused an increase in anchorage dependent growth (Figure 3c). A 3.4-fold increase in invasion was observed for CFPAC-1-HK2 relative to the control while a 2.4-fold increase was observed for PANC-1-HK2 (*P*<0.002, Figure 3e). Our data suggests that increased HK2 expression is sufficient to promote anchorage dependent and independent growth, as well as invasion.

### HK2 promotes invasion by regulating lactate production

**Figure 4:**
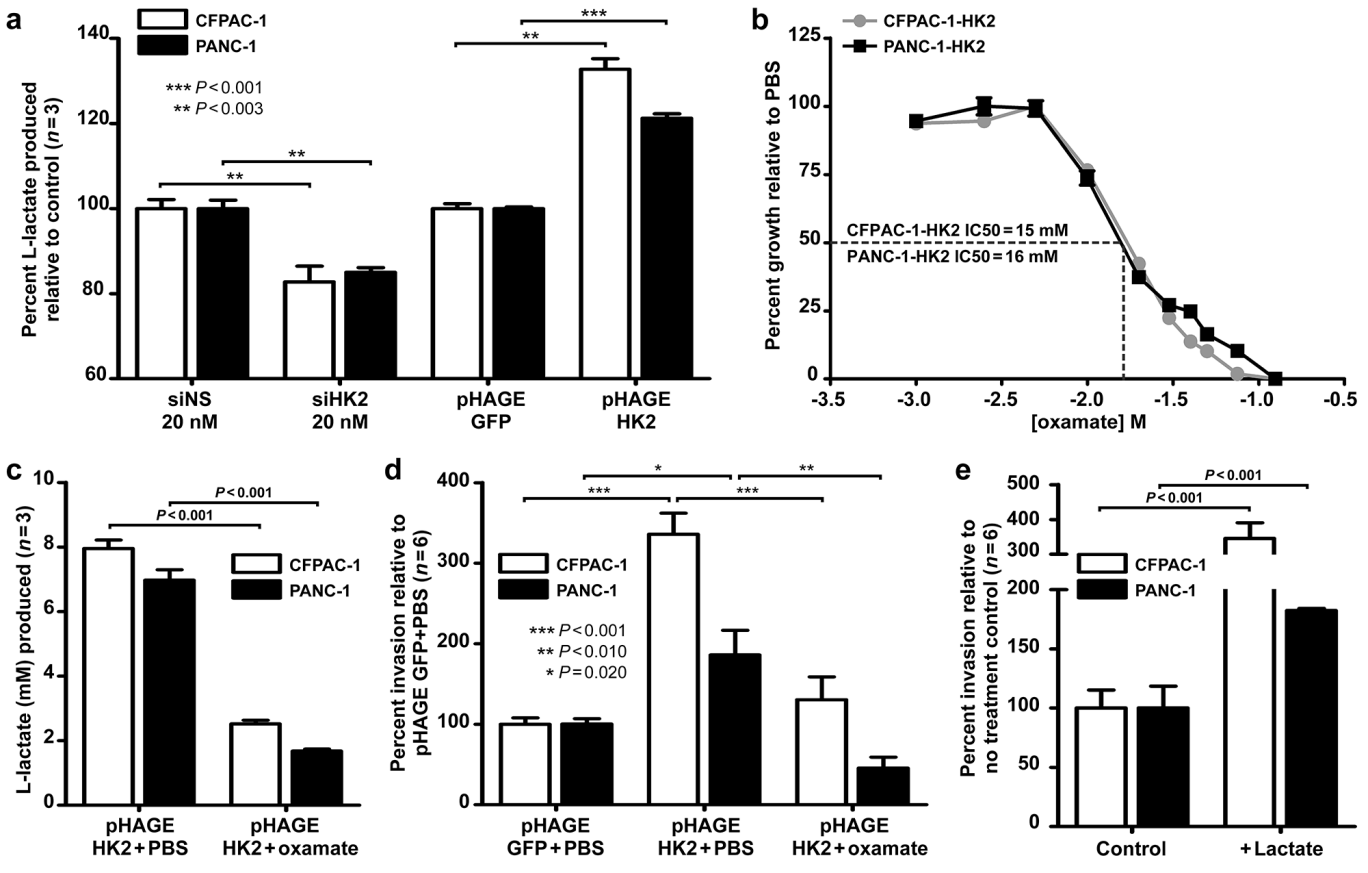
HK2 regulates lactate production and invasion in PDAC cell lines **a.** Relative lactate production in CFPAC-1 and PANC-1 with HK2 knockdown (siHK2 vs. siNS) and overexpression (pHAGE HK2 vs. pHAGE GFP). Mean ± SEM of biological replicates (*n*=3) shown with student's t-tests for statistical significance. **b.** IC50 determination for CFPAC-1-HK2 and PANC-1-HK2 after 72 hours of oxamate treatment; Mean ± SEM of technical replicates (*n*=4) shown with student's t-tests for statistical significance. CFAPC-1 IC50 calculated to be 15 mM while PANC-1 determined to be 16mM using GraphPad Prism software (v.5, GraphPad Software, INC. La Jolla, CA, USA). **c.** L-lactate produced (mM) in CFPAC-1-HK2 and PANC-1-HK2 cell lines treated with PBS or IC50 oxamate for 72 hours. Mean ± SEM of biological replicates (*n*=3) shown with student's t-tests for statistical significance. **d.** Percent invasion in cells treated with PBS or IC50 oxamate. Mean ± SEM of biological replicates (*n*=6) shown with student's t-tests for statistical significance. **e.** Percent invasion in PDAC cells incubated with extracellular lactate (40 mM). Mean ± SEM of biological replicates (*n*=6) shown with student's t-tests for statistical significance.

We hypothesized that the HK2-driven changes in invasion observed result from changes in glycolysis, as elevated glycolysis has been previously linked to metastasis [21, 22]. To this end, we measured lactate production in cell lines with transient knockdown and stable overexpression of HK2. “To ensure that changes in lactate production were a result of changes in the levels of HK2 and not a change in growth rate for the different experimental conditions examined, we measured lactate production over 24 hours after seeding an equal number of cells into the assay. At this time point no changes in proliferation or cell densities were observed (Supplementary Figure S2 and Figure 3c). An approximate 20% decrease in lactate production was observed with HK2 knockdown relative to the NS control (*P*<0.003, Figure 4a). Conversely, stable HK2 overexpression produced a 1.3-fold and 1.2-fold increase in lactate production for the CFPAC-1-HK2 and PANC-1-HK2 cell lines, respectively, suggesting that changes in HK2 are sufficient to alter glycolysis in PDAC cell lines (*P*<0.003, Figure 4a). To determine if HK2 promotes invasion in a lactate dependent manner, the pharmacologic inhibitor oxamate, a structural analog of pyruvate, was used to inhibit glycolysis in cells with stable HK2 overexpression. Cells were pretreated with oxamate at the calculated IC50 prior to seeding into transwell invasion assay (Figure 4b). At the time of seeding, a 3.2 and 4.1-fold decrease in lactate production was observed with oxamate treatment in CFPAC-1-HK2 and PANC-1-HK2, respectively, confirming inhibition of glycolysis relative to control (*P*<0.001, Figure 4c). An approximate 2.0-fold decrease in invasion was observed in oxamate treated cells with stable overexpression of HK2, suggesting that glycolysis is required for HK2 to promote invasion (*P*<0.010, Figure 4d). We next determined if the addition of extracellular lactate was sufficient to promote invasion. Cells were incubated with media supplemented with lactate (40 mM) for 24 hours, conditions that induce changes in histone acetylation and gene expression [30]. The addition of lactate was sufficient to promote invasion in both cell lines, as a 3.5-fold increase in invasion for CFPAC-1-HK2 and 2.5-fold increase in invasion of PANC-1-HK2 were observed (*P*<0.001, Figure 4e). These results suggest that HK2 regulates invasion in a lactate-dependent manner, supporting a direct link between elevated rates of glycolysis and increased metastatic potential.

### HK2 is necessary for PDAC tumor growth and promotes changes in gene expression *in vivo*

**Figure 5:**
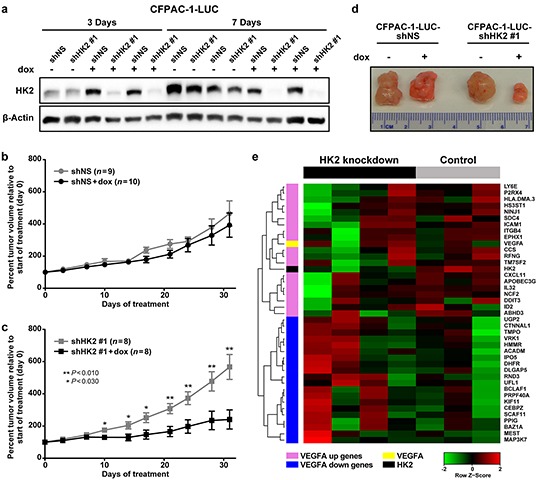
HK2 is required for PDAC primary tumor growth and regulates gene expression **a.** Expression of HK2 in CFPAC-1-LUC tumors containing doxycycline inducible shNS or shHK2#1 treated with sucrose (control) or doxycycline for 3 or 7 days. **b.** Percent tumor volume relative to start of treatment for shNS tumors treated with sucrose (black, *n*=9) or doxycycline (grey, *n*=10) for 30 days. Average normalized tumor volume and SEM shown. **c.** Percent tumor volume relative to start of treatment for shHK2 tumors treated with sucrose (black, *n*=8) or doxycycline (grey, *n*=8) for 30 days. Average normalized tumor volume and SEM shown with student's t-tests for statistical significance at each time point. **d.** Representative images of tumors isolated after 30 days of treatment with sucrose or doxycycline. **e.** Heat map of the expression of the top 20 ranked genes from the gene lists by Schoenfeld, et al [25] [32], including HK2 and VEGF-A, for shNS (control) and shHK2#1 (HK2 knockdown) tumors isolated at the end of treatment as determined by RNA-sequencing.

To examine the effects of HK2 knockdown on primary tumor growth, CFPAC-1-LUC cells containing a doxycycline-inducible shHK2#1 or shNS (control) were subcutaneously injected into immune-compromised mice. HK2 knockdown was confirmed in tumors expressing the shHK2#1 compared to those expressing shNS after 3 and 7 days of doxycycline administration (Figure 5a). To assess the effect of HK2 knockdown on long-term tumor growth, mice were given doxycycline or sucrose (control) once tumors reached an average volume of 152 mm^3^ (standard deviation (SD) 46 mm^3^). No change in growth was observed in the shNS tumors in mice treated with doxycycline, confirming that administration of doxycycline alone had no effect on tumor growth (Figure 5b). However, induction of shHK2#1 expression resulted in tumor growth inhibition during the 30 day treatment period compared to the shNS controls (*P*<0.030, Figure 5c). At the end of treatment an average reduction in tumor volume of 57.5% was observed with HK2 knockdown (*P*=0.020, Figure 5d).

RNA sequencing was performed on tumors harvested at the end of the study to determine the effect of HK2 knockdown on gene expression *in vivo*. Genes whose average reads per kilo base of transcript per million mapped reads (RPKM) was <10 were excluded from analysis, so that only genes with a high level of baseline expression were included (Supplementary Table S2, *n*=6,120). Gene set enrichment analysis (GSEA) [25] was performed to identify differentially regulated gene sets between control (shNS, *n*=3) and HK2 knockdown (shHK2#1,*n*=4), with a focus on gene sets contained in the molecular signature database's (MSigDBv5) hallmark and oncogenic signatures gene lists [25]. We found 27 gene sets to be significantly enriched in shNS tumors relative to shHK2 tumors (Supplementary Table S3, *P*<0.02, false discovery rate (FDR)<0.100) and 18 gene sets significantly enriched with HK2 knockdown relative to shNS (Supplementary Table S4, *P*<0.02, FDR<0.100) [25]. Interestingly, one of the highest enriched pathways in the control tumors included genes upregulated with increased vascular endothelial growth factor-A (VEGF-A) activity (VEGF_A_UP.VI_UP, normalized enrichment score (NES) 2.2, *P*<0.001, FDR<0.001, Figure 5e), a pathway important for promoting metastasis [31, 32]. Furthermore, genes downregulated with VEGF-A signaling were enriched in the HK2 knockdown tumors (VEGF_A_UP.VI_DN, NES -2.4, *P*<0.001, FDR<0.001, Figure 5e), suggesting that HK2 knockdown is associated with inhibition of VEGF-A signaling [32]. To validate the findings observed by RNA-sequencing, we examined the expression integrin β4, one gene known to regulated in a VEGF-A dependent manner [32], in tumors with HK2 knockdown (Supplementary Figure S3). We observed a decrease in total integrin β4 expression with HK2 knockdown, further supporting a link between VEGF-A signaling and HK2 expression.

### HK2 is required for PDAC metastasis *in vivo*

**Figure 6:**
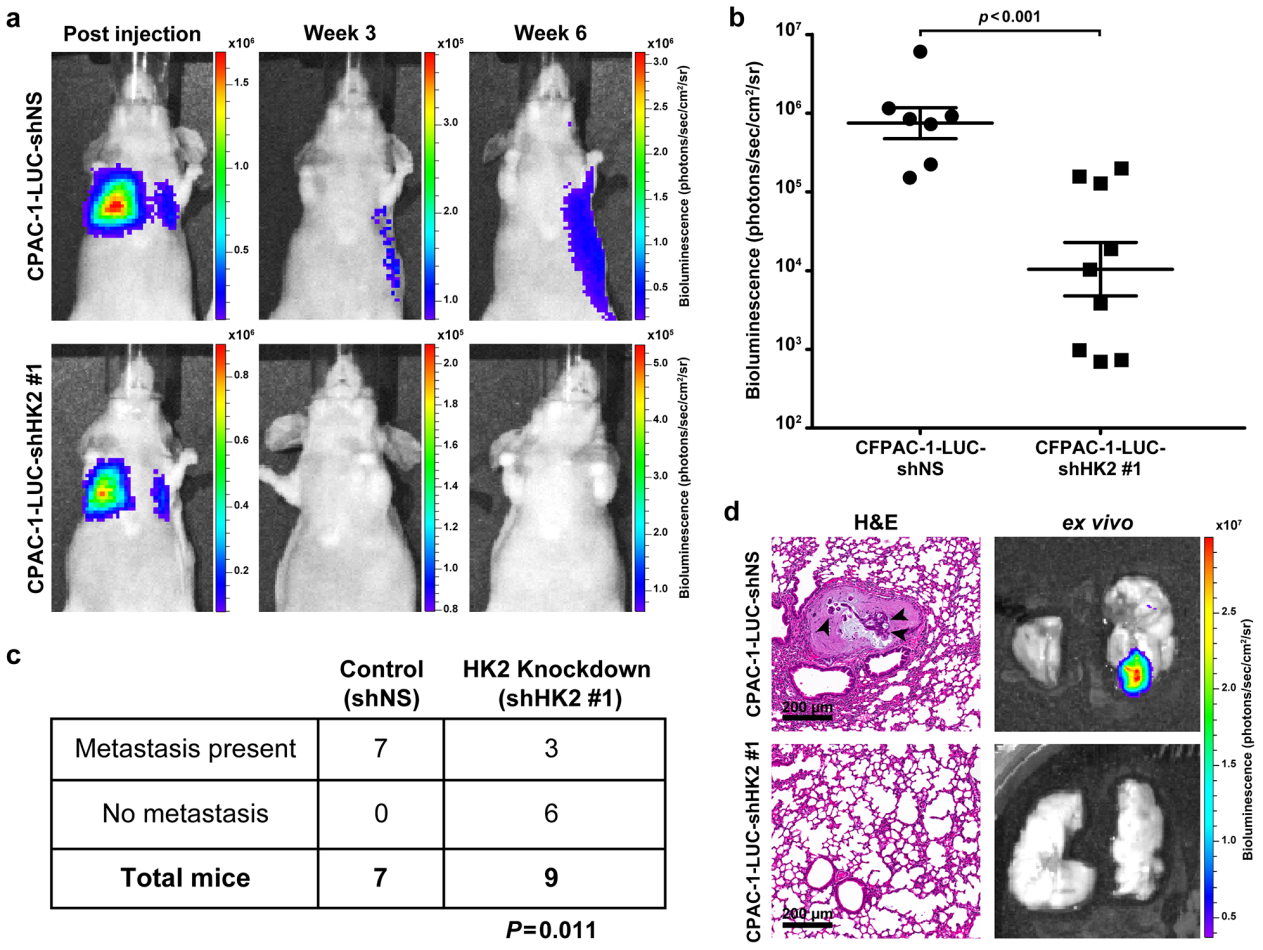
HK2 is required for metastasis in PDAC **a.** Bioluminescence of mice injected with CFPAC-1-LUC shNS cells (top row) and cells with HK2 knockdown (CFPAC-1-shHK2 #1, bottom row) at the start of the study (column one) and at the end of the study (column 3). **b.** Bioluminescence measured for tumors observed in lungs obtained after autopsy. Each point represents luminescence of tumor foci (shNS, *n*=7 and shHK2, *n*=3) or an entire lung if no foci were observed (shHK2, *n*=6). **c.** Fisher's exact t-test showing a significant difference in formation of metastases with HK2 knockdown (*P*=0.011). **d.** Representative *ex vivo* images used for quantification of bioluminescence. H&E staining was used to confirm metastases formation. Arrowheads point to cancer cells in a metastatic lesion with surrounding normal lung tissue (top row). Lungs that did not exhibit bioluminescence showed no histological evidence of metastases (bottom row).

To examine the requirement of HK2 for the promotion of metastasis, a tail vein assay was used [33]. CFPAC-1-LUC cells containing inducible shRNA constructs were treated with doxycycline prior to tail vein injection to induce shRNA expression (Figure 2a). Bioluminescence was used to monitor development of lung metastases and quantify tumor growth (Figure 6a). The average bioluminescence measured in the lungs of shNS injected mice was approximately 100-fold higher than that observed for the shHK2#1 injected mice (*P*=0.001, Figure 6b). The presence of metastatic disease was observed in 7 out of 7 mice injected with the shNS control compared to 3 out of 9 mice injected with shHK2#1 cells (*P*=0.011, Figure 6c). The presence of metastatic tumors was confirmed using hematoxylin and eosin (H&E) staining of sectioned lung tissue obtained from mice injected with shNS (Figure 6d, top row). No histological evidence of tumor formation was observed with injection of HK2 knockdown cells (Figure 6d, bottom row). These results show a requirement for HK2 in the extravasation and survival of cancer cells at distant organ sites, confirming an important role for HK2 in promoting metastasis.

## DISCUSSION

PDAC is a highly lethal disease with an increased incidence of metastasis and an overall poor prognosis [2]. Similar to a GEMM which showed genes regulating glucose metabolism to be upregulated in PDAC [8], we saw increased expression of key glycolytic genes, including GLUT1, HK2 and LDHA, in primary PDAC tumors relative to normal pancreas, suggesting a role for these genes in human tumorigenesis. We show that knockdown of HK2 results in decreased PDAC growth *in vitro* and *in vivo*, in agreement with what has been observed in other cancers [12, 16–18]. HK2 regulates glucose uptake, a process upstream of metabolic pathways including glycolysis, hexosamine biosynthesis, the pentose phosphate pathway and the citric acid cycle [4, 8]. Direct targeting of HK2 can, therefore, impede the flow of glucose into multiple downstream pathways necessary for KRAS driven tumor growth [8, 21]. Genetic deletion of HK2 in a preclinical model of Kras driven lung cancer was sufficient to alter glucose metabolism and improve overall survival [12], suggesting that direct targeting of HK2 would be beneficial in PDAC.

We also show that increased expression of HK2 in primary tumors is associated with shorter overall survival in PDAC patients undergoing curative surgery, in agreement with a smaller study that correlated increased HK2 protein expression with worse PDAC patient outcomes [19]. This, along with the observation that HK2 is upregulated in PDAC metastases relative to primary tumors, supports a role for HK2 in the metastatic process. Associations between increased HK2 expression, metastatic disease, and poor clinical outcomes have been observed in other cancers however a direct link between HK2 and metastasis has not been shown [19, 34, 35].

Here we provide direct evidence that HK2 is necessary and sufficient to promote metastasis in PDAC, as its increased expression promotes invasion and its knockdown inhibits cancer cell extravasation and colonization at distant organ sites, important components of the metastatic cascade [31, 36]. A study of PDAC cell lines correlated increased rates of glycolysis with aggressive tumor biology, suggesting that glycolysis may be important for metastasis [21]. We found that HK2 directly promotes metastasis via regulation of glycolysis, as pharmacologic inhibition of lactate production prevents HK2-driven invasion and extracellular lactate is sufficient to enhance invasion. This result is in agreement with previous studies showing extracellular lactate enhances migration of breast cancer cell lines, encourages metastases seeding of breast cancer cell lines *in vivo* and promotes motility of glioblastoma cell lines [37, 38]. As increased lactate production has also been linked to poor patient outcomes [21, 37, 39, 40]] we suggest that direct targeting of HK2 or inhibition of glycolysis may improve patient outcomes by limiting the formation of metastases.

Increased rates of glycolysis can promote invasion by altering the pH of the tumor microenvironment, enhancing cell signaling, influencing matrix metalloprotease activity and regulating gene expression [30, 37, 38, 41–43]. We show that genes involved in VEGF-A signaling, a pathway important for angiogenesis and metastasis [31], are significantly altered by HK2 knockdown. While a direct link between HK2 and VEGF-A was not assessed here, others have shown that lactate production influences VEGF-A signaling [42, 44, 45]. We show that extracellular lactate enhances PDAC cell invasion when present at concentrations known to influence gene expression [30]. We hypothesize that HK2 regulates lactate production and this, in turn, promotes VEGF-A signaling and changes in gene expression necessary for metastasis. While our data supports this hypothesis, more studies are needed to confirm the connection between HK2 and VEGF-A.

In conclusion the present study supports a requirement for HK2 in PDAC tumorigenesis and metastasis that helps explain the associated findings of high HK2 expression in PDAC patients with metastatic disease. We show that HK2 influences the invasive potential of PDAC cells by directly regulating glycolysis and that its knockdown induces changes in gene expression of pathways important for promoting metastasis, including VEGF-A signaling. Our data suggests that the targeting of HK2 may be a promising approach for treating metastatic PDAC.

## MATERIALS AND METHODS

### Cell culture and stable cell line generation

Human cell lines CFPAC-1, HPAF-II, Hs 766T, T3M4, and PANC-1 were obtained from the American Type Culture Collection and authenticated via short–tandem repeat profiling (Genetica, Burlington, NC, USA). The HPNE cell line was described previously [46]. CFPAC-1 was cultured in RPMI 1640, CFPAC-1-LUC (pLENTI-CMV-Luciferase) was cultured in RPMI 1640 with geneticin (500 μg/mL, Invitrogen, Grand Island, NY, USA), and PANC-1 was cultured in DMEM. All were supplemented with 10% fetal bovine serum (FBS, Invitrogen) and penicillin/streptomycin (Invitrogen) and incubated at 37°C in 5% CO2 atmosphere. For HK2 overexpression, the HK2 cDNA sequence from the pDONR-223-HK2 donor vector [47] (Addgene #23854) was cloned into a pHAGE puro destination vector (donated by the laboratory of William Kim, MD) using LR-clonase reaction as per the manufacturer's instructions (ThermoFisher Scientific #11791, Grand Island, NY, USA). For HK2 knockdown cell lines, shRNA sequences (below) were cloned into the pTRIPZ plasmid using EcoR1 and Xho1 restriction enzyme digestion of the pTRIPZ-shNS vector (donated by the laboratory of Channing Der, PhD). All constructs were verified by Sanger sequencing (Eton Biosciences, Research Triangle Park, NC, USA).

shHK2#1: CCGTAACATTCTCATCGATTT

shHK2#2: GCTACAAATCAAAGACAAGAA

A replication-incompetent lentivirus was generated in 293T cells using psPAX2 (Gag, Pol, Rev, Tat), pMD2.G (VSV-G), and target vector. For transduction, 1×10^6^ cells were seeded in 100 mm plates with media containing lentivirus and polybrene (8 μg/mL, Invitrogen). After 24 hours, infected cells were selected with puromycin (2 μg/mL, Invitrogen). To induce shRNA expression in pTRIPZ lines, cells were incubated for 72 hours with media containing 2 μg/mL doxycycline (Sigma-Aldrich, St. Lois, MO, USA).

### Transient knockdown with siRNA

Reverse transfection in a six-well plate was performed with Lipofectamine RNAiMax (Invitrogen) as per the manufacturer's guidelines. 4-5×10^5^ cells were seeded per well and a final concentration of 20 nM siNS (#4404021, ThermoFisher Scientific) or siHK2 (Catalog #S6560, Applied Biosystems, Grand Island, NY, USA) was used. Cells were incubated for 48 hours prior to use in assay or western blot.

### Western blot

Samples were lysed in 200 μL RIPA buffer (pH 7.4) containing protease inhibitors (ThermoFisher Scientific). 20-25 μg protein suspended in SDS loading buffer was run on 10% SDS polyacrylamide gels and electrotransferred to PVDF membranes. Membranes were blocked in 5% milk and incubated with 1:1,000 dilutions of primary antibodies in 5% BSA, including anti-HK1 (sc-#46695, Santa Cruz Biotechnology, Dallas, TX, USA), anti-HK2 (Catalog #2867, Cell Signaling Technology, Danvers, MA, USA), anti-integrin β4 (Cell Signaling Technology, Cat# 4707) and the loading controls anti-β-Actin (sc-#47778, Santa Cruz Biotechnology) and anti-vinculin (Catalog #V9131, Sigma). Membranes were incubated with 1: 5000 dilutions of appropriate secondary antibodies in 5% milk (ThermoFisher Scientific). Incubations were for 1 hour at room temperature and Clarity Western ECL substrate with ChemiDoc XRS+ imaging system (Bio-Rad Laboratories, Hercules, CA, USA) were used to detect immunoreactive bands.

### AIG

1-2 x10^4^ cells were seeded into a soft agar assay as was previously outlined in Martin et al [48]. Briefly a six well plate was coated with 2 mL of a 0.6% bacto-agar and culture medium mixture. 500 μL of a cell and 0.4% bacto-agar mixture was added after solidification of the first layer. 300 μL media was added every four days for two-three weeks. If cells were treated with doxycycline prior to seeding, administration of media with doxycycline (2 μg/mL) continued. Colony growth was using Image J software (NIH, Bethesda, Maryland, USA). Percent growth was calculated by dividing the number of colonies observed by the average number of colonies in the corresponding control.

### Transwell invasion

Uncoated inserts with 8-μm pores (Catalog #82050, Greiner Bio-One, Monroe, NC, USA) were coated with 100 μL of a diluted growth factor reduced Matrigel membrane matrix (300 μg/mL, ThermoFisher Scientific) and incubated at 37°C for 2 hours. Coated inserts were then placed into a 24 well plate containing 750 μL normal culture media. 1-2 x10^5^ cells were suspended in 250 μL media supplemented with 1% FBS and seeded into the upper chamber of insert. Cells invaded for 16 hours. Inserts were then cleaned, fixed, and stained with Diff Quik as per manufacturer's instructions (ThermoFisher Scientific). The number of cells invading was determined by counting five random fields per insert (counted by a blinded second party). Percent invasion was calculated by dividing the total number of cells invaded by the average number of cells invaded for the appropriate control.

### Hexokinase activity and lactate production

To examine hexokinase activity a colorimetric assay was performed as per manufacturer's instructions (Hexokinase activity kit, Catalog #MAK091, Sigma). Sample preparation included lysis of 1×10^6^ cells in assay buffer, with a 1:10 dilution of lysate used in assay. A glycolysis cell-based assay was performed to measure l-lactate production as per manufacturer's instructions (Catalog #600450, Caymen Chemical, Ann Arbor, MI, USA). 1×10^4^ cells in 200 μL were seeded per well in a 96-well plate for 24 hours. 20 μL of medium was collected into a new 96 well plate for colorimetric detection. Absorbance at appropriate wavelengths was measured with a Synergy 2 microplate reader (BioTek, Winooski, VT, USA). Percent hexokinase activity and lactate production were determined by dividing the corrected absorbance reading for each replicate by the average corrected absorbance for the appropriate control.

### Cell proliferation

To examine cell proliferation, 1×10^3^ cells in 200 μl were plated in quadruplicate into 96-well plates. After 24, 48, 72, or 96 hours of growth, 50 μl of 5 mg/mL 3-(4,5-dimethylthiazol-2-yl)-2,5-diphenyl tetrazolium bromide (MTT) dissolved in PBS was added to each well. After 30 minutes the mixture was aspirated and 200 μl of dimethyl sulfoxide (DMSO) was added to each well and mixed thoroughly. A_560nm_ was measured using a Synergy 2 microplate reader (BioTek). For IC50 determination 1×10^3^ cells in 200 μl were plated in quadruplicate into 96-well plates. After 24 hours, the medium was replaced with medium containing 75 mM PBS or oxamate (75 mM to 0.1 mM, Sigma). After 72 hour incubation, 50 μl MTT was added to each well and incubated for 30 minutes. The mixture was aspirated and 200 μl of DMSO was added to each well, mixed thoroughly, and A_560 nm_ was measured using a Synergy 2 microplate reader. The IC50 was calculated using GraphPad Prism software (v5, GraphPad Software, INC. La Jolla, CA, USA).

### Gene expression

#### qPCR

RNA was isolated from CFPAC-1, HPNE, HPAF-II, Hs 766T, T3M4, and PANC-1 cell pellets using an RNeasy Plus Mini Kit (Qiagen, Valencia, CA, USA). 2 μg of RNA was used for cDNA synthesis (Applied Biosystems) and 50 ng of RNA was used for real-time PCR (Applied Biosystems). Reactions were performed in triplicate on a 384 well plate using standard PCR settings on a QuantStudio 6 Flex Real-Time PCR system (Applied Biosystems). HK2 expression was assessed with HK2 TaqMan qPCR array (Applied Biosystems, Hs00606086_m1) while β-actin was assessed with ACTB TaqMan qPCR array (Applied Biosystems, Hs01060665_g1). The ΔΔCT method was used for analysis.

#### RNA sequencing

200-1000 ng of total RNA was used to prepare libraries with the TruSeq Stranded mRNA Sample Prep Kit (Illumina, San Diego, CA, USA). 75 bp paired-end reads were sequenced on a NextSeq 500 Desktop Sequencer using a high output flow cell kit (Illumina). Reads were separated by species of origin using Xenome [49]. Human specific reads were then aligned and quantified using Tophat2 [50], Cufflinks [51], hg19, mm10, and the UCSC transcript and gene definitions (genome.ucsc.edu). mRNA gene expression was analyzed with javaGSEAv2.2.1, and MSigDBv5.0 [25].

#### Animal Studies

All mouse studies were completed under protocols approved by the University of North Carolina at Chapel Hill's Institutional Animal Care and Use Committee.

#### Subcutaneous tumor injection

2×10^6^ CFPAC-1-LUC shRNA cells were subcutaneously injected into the flanks of 6-8 week old female nu/nu mice. To limit variability in induction of shRNA expression between control and experimental cell lines, cells containing doxycycline-inducible shNS were injected into the right flank while shHK2#1 cells were injected into the left flank of the same mouse. When tumors grew to a mean of 152 mm^3^ (SD 45.5 mm^3^) mice were randomized and given either 2.5% sucrose or 2.5% sucrose + 1 mg/mL doxycycline in drinking water for the specified time period. Tumor volume was measured three times per week and calculated using the formula (length×width^2^)/2. Student's t-tests compared the growth of treatment versus control during the study and a one-way ANOVA with Bonferroni correction for multiple comparisons test determined statistical significance of final tumor volume.

#### Tail vein injection

CFPAC-1-LUC shNS and CFPAC-1-LUC shHK2#1 cells incubated with culture medium containing 2 μg/mL doxycycline for 72 hours prior to injection. Treatment continued via administration of a 2.5% sucrose + 1mg/mL doxycycline throughout the study. 2×10^6^ cells in 100 μL PBS were injected into the tail veins of 6-8 week old female nu/nu mice as previously described in Elkin, et al [33]. Mice were monitored for lung metastases weekly after the initial injection using an IVIS Lumina Kinetic optical imaging system with an EMCCD camera (PerkinElmer, Waltham, MA, USA). Lungs were collected upon autopsy, fixed in 10% formalin, paraffin embedded (FFPE), and sectioned into 10 μM slices at 100 μM intervals and stained with H&E. Fisher's exact t-test determined statistical significance.

## SUPPLEMENTARY MATERIALS FIGURES AND TABLES




