# Supplementary material for: Hexokinase 2 promotes tumor growth and metastasis by regulating lactate production in pancreatic cancer
Source: Oncotarget. 2016 Jun 1;8(34):56081–94. doi: 10.18632/oncotarget.9760 (PMC5593546; doi:10.18632/oncotarget.9760)
Supplement: Supplementary file 1 [file oncotarget-08-56081-s001.pdf]

Hexokinase 2 promotes tumor growth and metastasis by regulating lactate production in pancreatic cancer

SUPPLEMENTARY MATERIALS

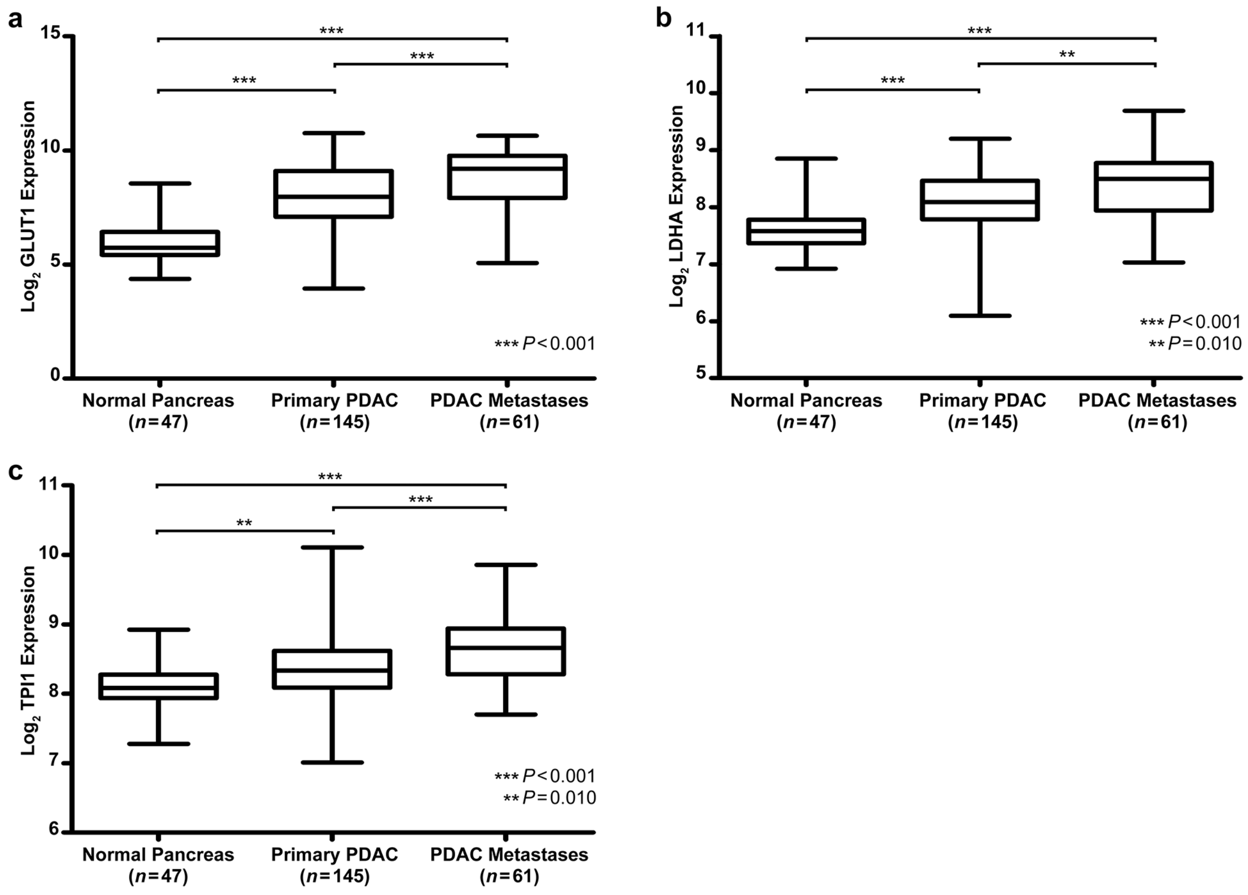

Supplementary Figure S1: Expression of a. GLUT1, b. LDHA, and c. TPI1 in normal pancreas, primary PDAC, and PDAC metastases.

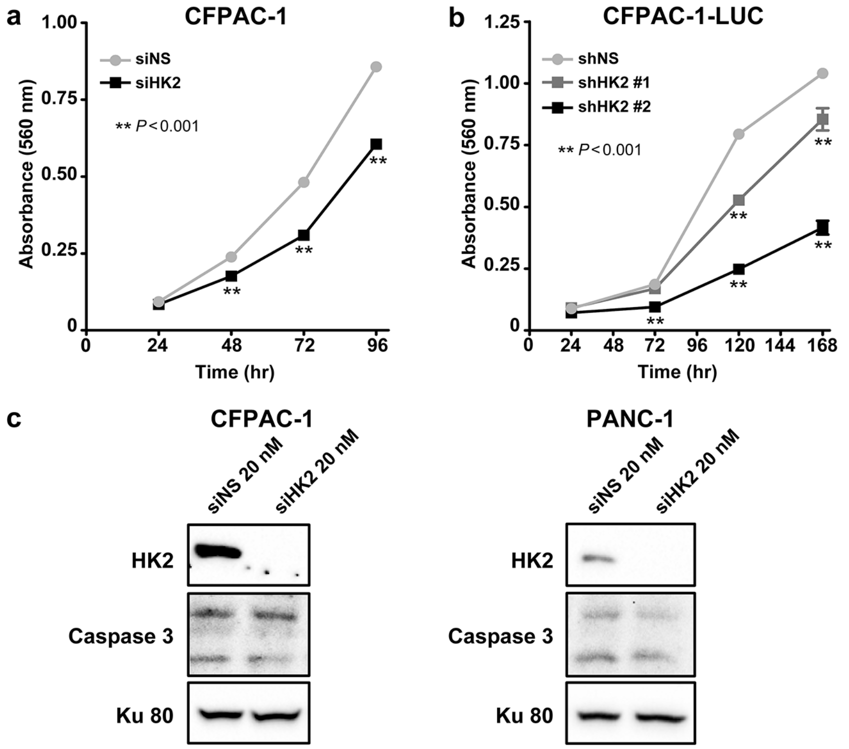

Supplementary Figure S2: HK2 regulates cell proliferation in PDAC. a. Anchorage dependent growth of CFPAC-1 cells with transient knockdown of HK2 (siHK2 20 nM) as determined using a MTT assay. Mean ± SEM of technical replicates (n=4) shown with student's t-tests for statistical significance at 48, 72, and 96 hours of growth. This growth trend was also observed in the PANC-1 cell line with transient HK2 knockdown. **b.** Anchorage dependent growth of CFPAC-1-LUC cells with stable knockdown of HK2 using shHK2#1 or shHK2#2 as determined using a MTT assay. Mean ± SEM of technical replicates (n=4) shown with student's t-tests for statistical significance at 72, 120, and 168 hours of growth for shHK2#1/2 relative to shNS. This growth trend was also observed in the PANC-1 cell line with stable HK2 knockdown. **c.** Expression of caspase 3 (top band, 35 kDa) and cleaved caspase 3 (bottom band, 17 kDa) in CFPAC-1 and PANC-1 cells with transient HK2 knockdown, where Ku 80 expression served as a loading control.

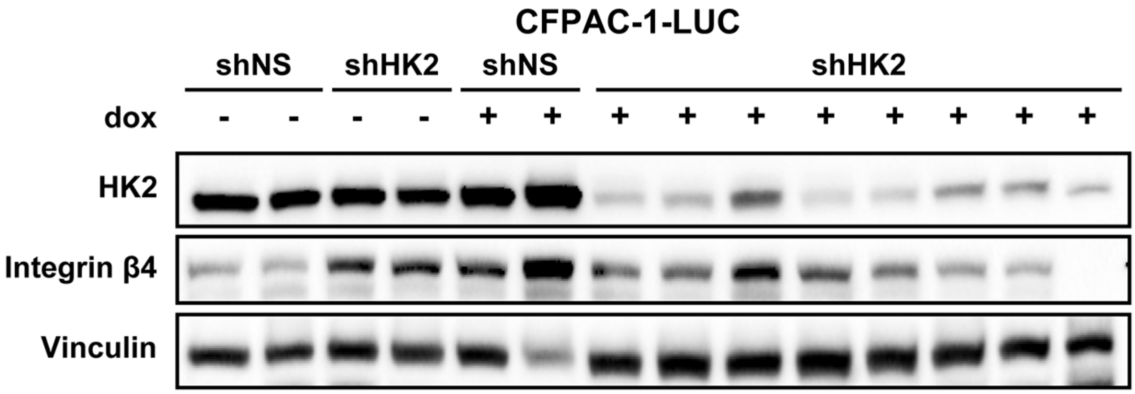

Supplementary Figure S3: Expression of integrin β4 is influenced by HK2 expression in PDAC primary tumors.

Supplementary Table S1: 153 unique genes included in the pentose phosphate pathway, O-glycan biosynthesis, glycolysis and gluconeogenesis, and glucose metabolism gene sets obtained from MSigDBv5 [25]

KEGG **PENTOSE PHOSPHATE PATHWAY**	***REACTOME*** **GLUCOSE METABOLISM**		***KEGG*** **O-GLYCAN BIOSYNTHESIS**	***KEGG*** **GLYCOLYSIS & GLUCONEOGENESIS**	
ALDOA	AGL	PFKL	B3GNT6	ACSS1	G6PC2
ALDOB	ALDOA	PFKM	B4GALT5	ACSS2	GALM
ALDOC	ALDOB	PFKP	C1GALT1	ADH1A	GAPDH
DERA	ALDOC	PGAM1	C1GALT1C1	ADH1B	GCK
FBP1	CALM1	PGAM2	GALNT1	ADH1C	GPI
FBP2	CALM2	PGK1	GALNT10	ADH4	HK1
G6PD	CALM3	PGM1	GALNT11	ADH5	HK2
GPI	ENO1	PGM2	GALNT12	ADH6	HK3
H6PD	ENO2	PHKA1	GALNT13	ADH7	LDHA
PFKL	ENO3	PHKA1P1	GALNT14	AKR1A1	LDHAL6A
PFKM	FBP1	PHKA2	GALNT2	ALDH1A3	LDHAL6B
PFKP	FBP2	PHKB	GALNT3	ALDH1B1	LDHB
PGD	GAPDH	PHKG1	GALNT4	ALDH2	LDHC
PGLS	GAPDHS	PHKG2	GALNT5	ALDH3A1	PCK1
PGM1	GBE1	PKLR	GALNT6	ALDH3A2	PCK2
PGM2	GOT1	PKM2	GALNT7	ALDH3B1	PDHA1
PRPS1	GOT2	PPP2CA	GALNT8	ALDH3B2	PDHA2
PRPS1L1	GPI	PPP2CB	GALNT9	ALDH7A1	PDHB
PRPS2	GYG1	PPP2R1A	GALNTL1	ALDH9A1	PFKL
RBKS	GYG2	PPP2R1B	GALNTL2	ALDOA	PFKM
RPE	GYS1	PPP2R5D	GALNTL4	ALDOB	PFKP
RPIA	GYS2	PRKACA	GALNTL5	ALDOC	PGAM1
TALDO1	MDH1	PRKACB	GALNTL6	BPGM	PGAM2
TKT	MDH2	PRKACG	GCNT1	DLAT	PGAM4
TKTL1	PC	PYGB	GCNT3	DLD	PGK1
TKTL2	PCK1	PYGL	GCNT4	ENO1	PGK2
	PCK2	PYGM	ST3GAL1	ENO2	PGM1
	PFKFB1	TPI1	ST3GAL2	ENO3	PGM2
	PFKFB2	TPI1P1	ST6GALNAC1	FBP1	PKLR
	PFKFB3	UGP2	WBSCR17	FBP2	PKM2
	PFKFB4			G6PC	TPI1

Supplementary Table S2: Expression of 6 120 genes in shHK2#1 (*n*=4) and shNS (*n*=3) in tumors obtained after 30 days treatment with doxycycline by RNA sequencing. This contains genes whose average RPKM was greater than 10 across all 7 samples examined.

See Supplementary File 1

Supplementary Table S3: Gene sets enriched in shNS tumors (*n*=3) relative to HK2 knockdown tumors (*n*=4)

Gene List	SIZE	NES	*P*-value	FDR
HALLMARK_INTERFERON_GAMMA_RESPONSE	133	2.61	*P* <0.001	0
HALLMARK_INTERFERON_ALPHA_RESPONSE	86	2.47	*P* <0.001	0
IL21_UP.V1_UP	29	2.17	*P* <0.001	3.11E-04
VEGF_A_UP.V1_UP	50	2.18	*P* <0.001	4.15E-04
KRAS.KIDNEY_UP.V1_DN	27	2.11	*P* <0.001	0.001
ALK_DN.V1_DN	31	1.97	*P* <0.001	0.004
HALLMARK_IL6_JAK_STAT3_SIGNALING	37	1.98	*P* <0.001	0.005
SINGH_KRAS_DEPENDENCY_SIGNATURE_	17	1.94	*P* <0.001	0.006
HALLMARK_COAGULATION	53	1.89	0.002	0.009
SRC_UP.V1_UP	35	1.82	0.002	0.017
PKCA_DN.V1_UP	27	1.79	0.000	0.020
KRAS.LUNG_UP.V1_DN	25	1.77	0.004	0.025
HALLMARK_P53_PATHWAY	117	1.73	0.000	0.032
WNT_UP.V1_UP	44	1.73	0.002	0.034
HALLMARK_REACTIVE_OXIGEN_SPECIES_ PATHWAY	35	1.73	0.006	0.035
KRAS.300_UP.V1_DN	23	1.74	0.008	0.036
BRCA1_DN.V1_DN	19	1.70	0.004	0.037
IL21_UP.V1_DN	22	1.69	0.015	0.038
HALLMARK_TNFA_SIGNALING_VIA_NFKB	118	1.67	0.002	0.045
KRAS.BREAST_UP.V1_UP	26	1.60	0.018	0.075
ATF2_UP.V1_UP	43	1.58	0.019	0.082
GCNP_SHH_UP_LATE.V1_DN	69	1.54	0.006	0.092
STK33_SKM_UP	93	1.53	0.012	0.094
ESC_J1_UP_EARLY.V1_DN	73	1.54	0.014	0.095
PRC1_BMI_UP.V1_UP	27	1.54	0.033	0.097
MEL18_DN.V1_DN	53	1.55	0.014	0.097
IL2_UP.V1_UP	49	1.54	0.016	0.098

This includes gene sets found to have a $P<0.020$ and an FDR<0.100. Gene sets analyzed were contained in the MSigDBv5 hallmark and oncogenic gene signature lists [25].

Supplementary Table S4: Gene sets enriched in tumors with HK2 knockdown (*n*=4) relative to shNS (*n*=3)

Gene List	SIZE	NES	*P* value	Q value
HALLMARK_MYC_TARGETS_V1	195	-2.51	*P* <0.001	0.000
HALLMARK_E2F_TARGETS	144	-2.35	*P* <0.001	0.000
VEGF_A_UP.V1_DN	108	-2.34	*P* <0.001	0.000
HALLMARK_SPERMATOGENESIS	27	-2.30	*P* <0.001	0.000
PIGF_UP.V1_UP	93	-2.27	*P* <0.001	0.000
GCNP_SHH_UP_LATE.V1_UP	98	-2.14	*P* <0.001	0.001
HALLMARK_G2M_CHECKPOINT	131	-2.09	*P* <0.001	0.001
CSR_EARLY_UP.V1_UP	90	-1.90	*P* <0.001	0.007
ERB2_UP.V1_DN	100	-1.84	*P* <0.001	0.011
GCNP_SHH_UP_EARLY.V1_UP	87	-1.74	*P* <0.001	0.025
HALLMARK_MTORC1_SIGNALING	168	-1.69	*P* <0.001	0.032
TBK1.DF_DN	146	-1.62	*P* <0.001	0.053
CORDENONSI_YAP_CONSERVED_SIGNATURE	35	-1.75	0.002	0.025
HALLMARK_MITOTIC_SPINDLE	100	-1.70	0.002	0.031
HALLMARK_MYC_TARGETS_V2	42	-1.66	0.004	0.038
E2F1_UP.V1_UP	90	-1.55	0.006	0.076
CSR_LATE_UP.V1_UP	109	-1.59	0.010	0.058
PRC2_EZH2_UP.V1_UP	51	-1.61	0.014	0.053

This includes only gene sets found to have a $P<0.020$ and an FDR<0.100. Gene sets analyzed were contained in the MSigDBv5 hallmark and oncogenic gene signature lists [25].
